# Dielectrophoretic Enrichment Coupled with Impedance Spectroscopy for Real-Time Bacterial Detection and Antibiotic Susceptibility Testing Using an Interdigitated Wave Electrode Array

**DOI:** 10.3390/s26154984

**Published:** 2026-08-06

**Authors:** Zeeshan, Naeem Iqbal

**Affiliations:** 1 School of Computing, Gachon University, Seongnam-si 13120, Gyeonggi-do, Republic of Korea; 2 Centre for Secure Information Technologies, Queen’s University Belfast, Belfast BT3 9DT, UK; n.iqbal@qub.ac.uk; 3 Momentum 1.0 Research, Queen’s University Belfast, Belfast BT3 9DT, UK

**Keywords:** dielectrophoresis (DEP), impedance spectroscopy (IS), interdigitated wave electrode array (IWEA), finite element modeling (FEM), antibiotic susceptibility testing (AST), Gram-positive and Gram-negative bacteria

## Abstract

Antimicrobial resistance (AMR), largely driven by the inappropriate and excessive use of antibiotics, requires rapid and reliable bacterial detection and antibiotic susceptibility testing (AST), as conventional culture-based methods remain time-intensive. Here, we report a real-time, label-free interdigitated wave electrode array (IWEA) that combines dielectrophoretic (DEP) enrichment with impedance spectroscopy for rapid bacterial detection and AST. The proposed IWEA was designed and optimized via COMSOL Multiphysics to enhance DEP-relevant electric-field strength, thereby improving DEP-assisted bacterial enrichment compared to conventional planar interdigitated electrodes. The platform enabled sensitive detection of both Gram-positive (*Staphylococcus aureus*) and Gram-negative (*Escherichia coli*) in 0.1× PBS across 10–10^5^ CFU/mL within 30 min using DEP-assisted preconcentration (100 kHz, 10 V_pp_), outperforming passive (non-DEP) operation (10^2^–10^5^ CFU/mL). For AST, bacterial responses to vancomycin, gentamicin, and ampicillin were monitored through impedance variations following DEP-based enrichment. Susceptible bacteria produced concentration-dependent reductions in ΔZ/Z_0_, whereas resistant bacteria showed similar responses to untreated controls. Quantitative assessment yielded CC_50_ values of 2.86 ± 0.22 µg/mL and 3.06 ± 0.20 µg/mL for vancomycin and gentamicin against *S. aureus*, and 4.59 ± 0.30 µg/mL for gentamicin against *E. coli*. Resistance profiles of *S. aureus* to ampicillin and *E. coli* to vancomycin and ampicillin were clearly distinguished. SEM imaging and disk diffusion assays independently validated the impedance-derived susceptibility results. Collectively, this IWEA platform offers a rapid, label-free, and quantitative approach for bacterial detection and AST, with strong potential for antimicrobial screening and point-of-care diagnostics.

## 1. Introduction

Bacterial infections remain a critical global health concern, contributing to over 8 million deaths annually worldwide and placing a substantial burden on healthcare systems [1]. The rapid emergence and spread of antimicrobial resistance (AMR) have further complicated clinical treatment [2], making timely pathogen identification and rapid antibiotic susceptibility testing essential for effective clinical management and for reducing infection-related mortality. Traditional bacterial detection methods such as cell culture, microscopy, and biochemical tests are time-consuming, labor-intensive, and require specialized facilities [3]. PCR-based molecular techniques improve speed and sensitivity but still depend on advanced instruments and reagents [4]. Similarly, conventional antibiotic susceptibility testing (AST) approaches, including disk diffusion, E-test, and broth dilution methods, determine bacterial susceptibility by assessing growth inhibition in response to antibiotic treatment [5,6,7]. Despite their reliability and accuracy, conventional AST methods are often time-consuming, requiring pure isolation, culture enrichment, and prolonged turnaround time of 24–48 h. More recently, several phenotypic approaches for rapid AST have emerged, including microscopy-based growth monitoring [8,9], density and biomass detection using microchannel resonance [10] and microcantilevers [11], field-assisted methods employing magnetic or electric manipulation [12,13], and viability assessment using fluorescent dyes [14] or 16s rRNA quantification techniques [15]. These methods typically reduce the overall assay time to approximately 3–8 h but still require extensive optimization, complex sample preparation, and additional pre-treatment steps.

To overcome these limitations and challenges, impedance-based electrochemical sensing approaches have gained significant attention for rapid and simple real-time bacterial detection [16,17,18,19]. The current bacterial impedance sensing platforms can be broadly categorized into two types. The first is based on monitoring metabolic activity, where changes in impedance arise from certain metabolites [20]. The second relies on detecting target binding events at the sensor surface, where impedance variations are induced by the interaction between analytes and immobilized bioreceptors such as antibodies [21,22]. The first type is often limited by reduced sensitivity and indirect signal generation, while the second type typically requires complex chip architectures and extensive electrode surface modification to enhance impedance signal output. Similarly, in antibiotic susceptibility testing (AST), impedance-based sensing enables real-time, label-free monitoring of bacterial physiological responses to antibiotic exposure [23,24,25,26]. Several recent studies have reported impedance-based viability assays that provide binary (yes/no) responses in the presence of antibiotics [27,28,29]. While such approaches can identify effective antibiotic-pathogen combinations through cell–drug interaction analysis, they are generally unable to generate concentration-dependent dose–response curves, such as minimum inhibitory concentration (MIC), which are typically obtained from conventional antibiogram methods [30]. This limitation can be attributed to the use of highly conductive culture media, such as Luria–Bertani broth, which elevate background signals and reduce impedance sensitivity. Additionally, the lack of precise spatial localization of bacterial cells within the electrode sensing region limits efficient electrode–cell interaction, thereby reducing the accuracy of electric-field-based sensing approaches.

The efficiency and accuracy of these biosensors are significantly enhanced through dielectrophoresis (DEP)-integrated impedance measurement, an active particle manipulation strategy that enables selective enrichment of bacterial cells at the sensing interface, thereby facilitating real-time bacterial detection and viability-dependent antibiotic susceptibility testing (AST) [31,32,33,34,35]. DEP refers to the motion of polarizable particles in a non-uniform electric field, a phenomenon first observed by Reuss and later investigated and formally termed dielectrophoresis by Pohl [36,37]. The dielectrophoretic force can be expressed as:

(1) FDEP=2πrp3εr ε0 ReKω∇E2

(2) $$K\left(\omega \right)=\frac{\varepsilon_{p}^{*}-\varepsilon_{m}^{*}}{\varepsilon_{p}^{*}+2\varepsilon_{m}^{*}}$$

where $F_{DEP}$ is the dielectrophoretic force in Newtons $\left(N=\mathrm{kg}.m/s^{2}\right)$, $r_{p}$ represents the particle radius (m), $\nabla E^{2}$ is the gradient of the square of the electric field ($V^{2}/m^{3}$), and Re[*K(ω)*] corresponds to the real part of the Clausius–Mossotti factor (CM), which is dimensionless. The term $\varepsilon_{r\;}$ is the relative permittivity of the surrounding medium (dimensionless), and $\varepsilon_{0\;}$ is the vacuum permittivity constant, defined as 8.85 × 10^−12^ [F/m]. Here, $\varepsilon_{p}^{*}$ and $\varepsilon_{m}^{*}$ are the complex permittivity’s of the particle and medium, respectively. Under an applied AC electric field, bacterial cells become polarized and experience dielectrophoretic (DEP) forces. Depending on whether the real part of the Clausius–Mossotti factor (Re[*K(ω)*]) is positive or negative, cells are either selectively concentrated near regions of high electric-field intensity (positive DEP) or move away toward the minimum field intensity region (negative DEP). This cell redistribution on the electrode surface alters the interfacial electrical properties, resulting in measurable impedance variations. The impedance of the system is determined from the ratio of the applied voltage to the measured current:

(3) Z=VI=R+1jωC

The performance of DEP-integrated impedance sensors can be precisely tuned by regulating key operational parameters, including electric-field strength, excitation frequency, and the electrical conductivity of the suspending medium. The non-uniform electric fields required for DEP generation are strongly governed by the geometry and spatial arrangement of electrodes within the sensing region, making electrode design a decisive factor in determining particle trapping efficiency and overall sensing performance. Recent advances in microfabrication have enabled the development of complex microelectrode arrays and microscale architectures, allowing precise modulation of electric-field gradients [38]. As a result, the design of the flow channel and electrode configuration offers multiple degrees of freedom for optimizing electrode geometries to produce high-electric-field gradients (∇*E*^2^) and achieve efficient and controlled particle manipulation. In parallel, nanomaterials have significantly advanced biosensing technologies by enhancing signal amplification, with graphene, carbon nanotubes, quantum dots, and metallic nanoparticles among the most widely utilized functional materials [39].

**Figure 1:**
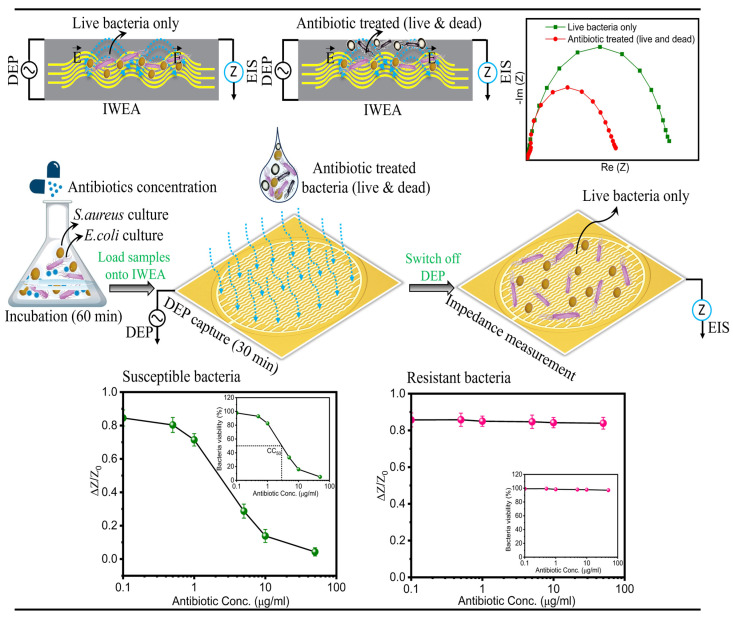
Schematic representation of the DEP-integrated impedance biosensing platform for rapid bacterial detection and AST. The workflow illustrates antibiotic treatment, sample loading, DEP-enrichment of viable bacteria, and impedance-based readout of susceptible and resistant bacteria.

In this work, we developed a DEP-integrated impedimetric interdigitated wave electrode array (IWEA) for rapid, label-free bacterial detection and antibiotic susceptibility testing (AST). The electrode geometry was optimized using COMSOL Multiphysics 5.6 to enhance electric-field localization and improve DEP-driven bacterial enrichment compared to conventional planar interdigitated electrodes. The optimized IWEA was fabricated on a printed circuit board, surface-modified with Pt-black to enhance electrochemical activity and functionalized with antibodies for selective bacterial capture. Using this platform, we demonstrate sensitive detection of both Gram-positive (*S. aureus*) and Gram-negative (*E. coli*) bacteria in 0.1× PBS over a wide concentration range (10–10^5^ CFU/mL) within 30 min using DEP-assisted preconcentration (100 kHz, 10 V_pp_), outperforming passive (non-DEP) operation. Furthermore, the IWEA enables real-time AST through impedance monitoring of antibiotic-induced bacterial viability changes. Susceptible bacteria exhibit a pronounced concentration-dependent response, while resistant bacteria remain similar to untreated controls. SEM imaging and disk diffusion assays independently validated the impedance-derived susceptibility results. Additionally, CC_50_ values (antibiotic concentration causing 50% reduction in viability) were extracted from impedance-based dose–response curves. The working mechanism of the developed IWEA is illustrated in Figure 1.

## 2. Experimental Methods

### 2.1. Reagents, Chemicals and Instrumentation

*S. aureus* (ATCC 12692) and *E. coli* (ATCC 43888) were used as target bacterial strains. Monoclonal antibodies against *S. aureus* (ab20920) and *E. coli* (ab35292) were purchased from Abcam (Cambridge, UK). Hydrogen hexachloroplatinate (IV) hexahydrate (H_2_PtCl_6_·6H_2_O, ≥37.5% Pt basis), hydrochloric acid (HCl), lead diacetate trihydrate (≥99%), 6-Mercapto hexanoic acid (90%), *N*-(3-(dimethylamino) propyl)-*N*-ethylcarbodiimide (EDC), *N*-hydroxysuccinimide (NHS, 98.0%), Glutaraldehyde, Potassium ferrocyanide (K_4_[Fe(CN)_6_]), Potassium ferricyanide (K_3_[Fe(CN)_6_]), Vancomycin, Gentamicin, and Ampicillin were obtained from Sigma-Aldrich (Seoul, Republic of Korea). Phosphate-buffered saline (PBS) and bovine-serum albumin (BSA) were purchased from Tech and Innovation (Shuncheon, Republic of Korea).

Electrochemical impedance spectroscopy (EIS) measurements were performed using an EmStat Pico potentiostat (EmStat Pico MUX16, PalmSens BV, Houten, The Netherlands; frequency range: 0.016 Hz–200 kHz). Surface morphology and topographical analyses were performed using a scanning electron microscope (SU8600, Hitachi, Tokyo, Japan; spatial resolution: 0.6 nm at 15 kV) and an atomic force microscope (XE-100, Park Systems, Suwon, Republic of Korea; XY resolution: 1.0 nm). An alternating current (AC) electric field was applied using a function generator (33500B, Keysight Technologies, Santa Rosa, CA, USA; frequency resolution: 1 µHz) for dielectrophoretic manipulation of bacterial cells. Bacterial concentrations were determined by measuring the optical density (OD) using a spectrophotometer (OPTIZEN POP, K LAB Co., Ltd., Daejeon, Republic of Korea; spectral bandwidth: 1.8 nm).

### 2.2. Bacterial Culture Preparation

The bacterial strains *Staphylococcus aureus* and *Escherichia coli* were cultured in liquid growth medium under optimal incubation conditions until the exponential growth phase was achieved. Following incubation, 100 ± 1.0 µL aliquots of each culture were analyzed at 600 nm using a spectrophotometer to determine bacterial concentration. After incubation, the cultures were centrifuged, washed with PBS to remove residual media components, and resuspended in fresh PBS. Serial dilutions were subsequently prepared to obtain working concentrations ranging from 10 to 10^5^ CFU/mL for subsequent experiments.

### 2.3. Antibiotic Susceptibility Tests

Cell viability in the presence of different antibiotics was evaluated using plating and impedance-based analysis. Three antibiotics, namely vancomycin, gentamicin, and ampicillin, were selected to evaluate susceptibility in *S. aureus* and *E. coli*. Initially, 700 ± 5.0 µL of bacterial suspensions were incubated with different concentrations of antibiotics ranging from 0.1 to 50 µg/mL for 1 h. Following incubation, the samples were centrifuged and washed with PBS to remove residual antibiotics and medium and subsequently resuspended in PBS for impedance analysis. In addition, disk diffusion assays were performed using BBL™ Sensi-Disc™ antimicrobial susceptibility test discs (BDs) to evaluate antibiotic susceptibility. Bacterial suspensions were uniformly spread onto agar plates using sterile cotton swabs. Antibiotic-impregnated discs containing vancomycin (10 μg/mL), gentamicin (10 μg/mL), and ampicillin (50 μg/mL) were then placed onto the agar surface, followed by incubation at 37 °C for 12–14 h. For SEM analysis, antibiotic-treated bacterial suspensions were centrifuged, washed with PBS, and fixed using 0.5% glutaraldehyde solution. The samples were subsequently dried prior to SEM imaging to investigate antibiotic-induced morphological changes in bacterial cells.

### 2.4. Finite Element Analysis of Electric-Field Distribution for DEP Applications

To guide the biosensor design for dielectrophoresis (DEP)-assisted bacterial capture and antibiotic susceptibility testing (AST), a finite element model (FEM) was developed using COMSOL Multiphysics 5.6. The model focused on evaluating geometry-dependent variations in electric-field distribution between a conventional planar interdigitated electrode array (IDEA) and an interdigitated wave electrode array (IWEA). In addition to visualizing the electric-field norm, the average squared electric-field magnitude (E^2^) was computed in the electrolyte domain. According to Equation (1), the dielectrophoretic force on a particle is proportional to the gradient of (E^2^); therefore, regions with higher and more spatially extended (E^2^) are expected to generate stronger DEP forces. The simulation model consisted of a counter electrode and a working electrode fabricated from gold (σ = 4.11 × 10^7^ S/m, ε = 1.14) [40], immersed in a phosphate-buffered saline (PBS) medium (σ = 0.16 S/m, ε = 80) representing the electrolyte environment [41]. All simulations were performed using the electric current interface in COMSOL Multiphysics 5.6, solved by the following equations.

(4) ∇⋅J=Q

(5) $$J=\sigma E+j\omega D+J_{e}\;$$

**Figure 2:**
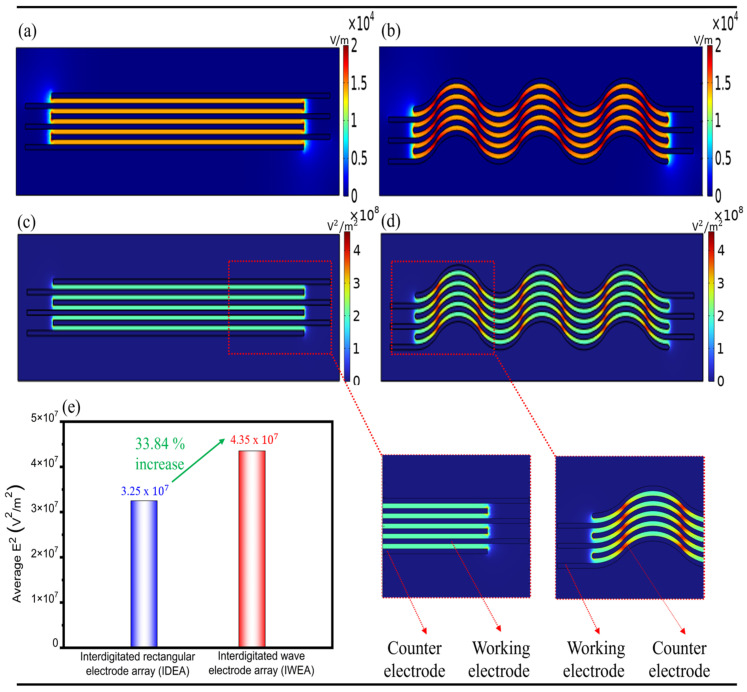
Finite element simulation of the electric-field norm and average squared electric field (E^2^) for the conventional interdigitated electrode array (IDEA) and the interdigitated wave-shaped electrode array (IWEA). (**a**) Electric-field norm of the IDEA. (**b**) Electric-field norm of the IWEA. (**c**) Average squared electric field (E^2^) of the IDEA. (**d**) Average squared electric field (E^2^) of the IWEA. (**e**) Relative enhancement in average squared (E^2^) electric field, demonstrating a 33.84% increase for the IWEA compared with the IDEA.

(6) E=−∇V 

where J denotes current density (A/m^2^), Q is the electric charge source density (A/m^3^), σ stands for electrical conductivity (S/m), E refers to electric field (V/m), ω represents angular frequency (rad/s), D defines electric displacement (C/m^2^), $J_{e}\;$ indicates external current density (A/m^2^), and V corresponds to electric potential (V). The simulated electric-field distributions for the IDEA and IWEA geometries are presented in Figure 2a,b, respectively. The conventional IDEA exhibited a highly non-uniform electric-field distribution, with strong field localization occurring predominantly at the electrode edges and comparatively weaker fields in the central gap regions. In contrast, the IWEA generated a more spatially extended and continuous high-field region throughout the sensing area. This behavior is further reflected in the (E^2^) maps, which show higher (E^2^) regions between the IWEA compared to the conventional IDEA, indicating stronger and more spatially distributed field intensity in the IWEA design, as shown in Figure 2c,d. To quantify these differences, the average squared electric field (E^2^) was evaluated in the electrolyte region for both IDEA and IWEA geometries. The IWEA exhibited a higher average square electric field (E^2^ = 4.35 × 10^7^ V^2^/m^2^) compared to the IDEA (E^2^ = 3.25 × 10^7^ V^2^/m^2^), corresponding to an increase of 33.84%, as shown in Figure 2e. This higher average E^2^ confirms that the interdigitated wave electrode array (IWEA) generates a stronger electric field environment for DEP-based manipulation, supporting its selection for fabrication and experimental validation.

### 2.5. Fabrication of IWEA

**Figure 3:**
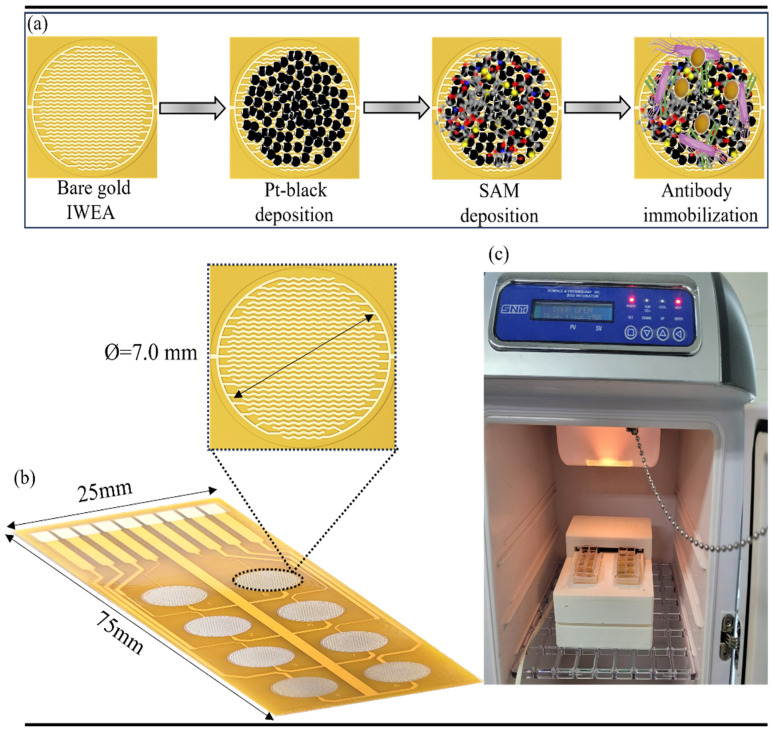
(**a**) Schematic illustration of the stepwise fabrication process of the IWEA biosensor. (**b**) Optical image of the fabricated IWEA platform comprising eight working/sensing electrodes and a common counter electrode. (**c**) Optical image of the complete experimental setup, including the IWEA and a custom-designed holder for bacterial incubation.

The interdigitated wave electrode array (IWEA) was manufactured by CELLAMES, (Seongnam, Republic of Korea) on a printed circuit board (PCB), as shown in Figure 3b. Each array comprises eight individual working (sensing) electrodes with a common counter electrode. The fabrication process involved sequential deposition of nickel (3 µm) followed by gold (0.8 µm) onto a 1 mm thick PCB substrate using electron-beam evaporation. Both the working and counter electrodes were designed with a uniform finger width and spacing of 100 µm. The overall PCB dimensions of 75 mm × 25 mm were used to accommodate the IWEA structure, with each sensing array providing a circular active detection region of 7.0 mm diameter.

A custom-designed 3D-printed holder was fabricated for bacterial incubation and electrochemical measurements, as illustrated in the experimental setup in Figure 3c. This platform is designed to hold two IWE arrays, allowing parallel operation of up to 16 independent electrodes. It features separate micro-wells positioned over each sensing region, creating isolated chambers that enable precise sample introduction, incubation, and washing under controlled conditions. Electrochemical measurements were performed using an EmStat Pico potentiostat, enabling multiplexed sequential readout across 16 independent sensing channels.

### 2.6. Preparation of IWEA Biosensor

The bare IWEA was first cleaned using ethanol and deionized water to eliminate any surface contaminants, followed by nitrogen drying and oxygen plasma treatment. To enhance the electrochemical activity of the IWEA, Pt-black was electrodeposited onto the electrode surface, forming a highly porous nanostructured layer. The pt-black deposited IWEA was cleaned and further functionalized with 6-mercaptohexanoic acid solution (100 mM) for 12 h, followed by activation of carboxyl groups using EDC/NHS for 15 min at room temperature. Following surface activation, the IWEA was exposed to 0.5 mg/mL *S. aureus* antibody solution for 1 h, enabling covalent attachment of antibodies to the sensing surface. The same protocol was employed for *E. coli* antibody immobilization. To block nonspecific adsorption, the IWEA surface was further treated with 0.5% BSA, thereby completing the fabrication of the biosensing platform as shown in Figure 3a.

### 2.7. Electrochemical Measurements

Electrochemical impedance spectroscopy (EIS) was performed using the IWEA platform mounted in a custom 3D-printed holder and interfaced with an EmStat Pico potentiostat. For bacterial detection, 700 ± 5.0 µL of suspensions (10–10^5^ CFU/mL) were introduced onto the IWEA. In DEP-assisted measurements, an AC electric field (100 kHz) at 10 V_pp_ was applied for 30 min to concentrate bacteria at the IWEA surface before impedance acquisition, whereas passive or non-DEP measurements were carried out without electric-field exposure after incubation only. For AST, *S. aureus* and *E. coli* suspensions (10^5^ CFU/mL) were incubated for 1 h with vancomycin, gentamicin, and ampicillin. After incubation, the treated samples were loaded onto the IWEA platform and subjected to DEP enrichment for 30 min to selectively concentrate viable cells at regions of high electric-field intensity. Following DEP enrichment, the electric field was turned off, and impedance measurements were immediately performed at a low excitation voltage of 50 mV to monitor antibiotic-induced changes in bacterial viability. Results were expressed as differential impedance response (ΔZ) relative to the initial impedance at t = 0 and reported as a function of frequency. Each experiment was repeated three times.

## 3. Results and Discussions

### 3.1. Pt-Black-Modified IWEA: Surface Morphology and Electrochemical Properties

Pt-black was electrodeposited onto the interdigitated wave electrode arrays (IWEA) to enhance the electrochemical activity and sensing capability of the biosensor platform. The deposition process was carried out within the sensing region using a conventional three-electrode setup in an electrolyte composed of hydrogen hexachloroplatinate (IV) hexahydrate (2.5%), lead diacetate trihydrate (0.05%), and 0.01 M HCl. To optimize the Pt-black nanostructure, a constant current density of −2.5 mA cm^−2^ was applied for different deposition durations (120 s, 240 s, and 400 s), enabling systematic control over the surface morphology and electrochemical characteristics of the modified electrodes.

**Figure 4:**
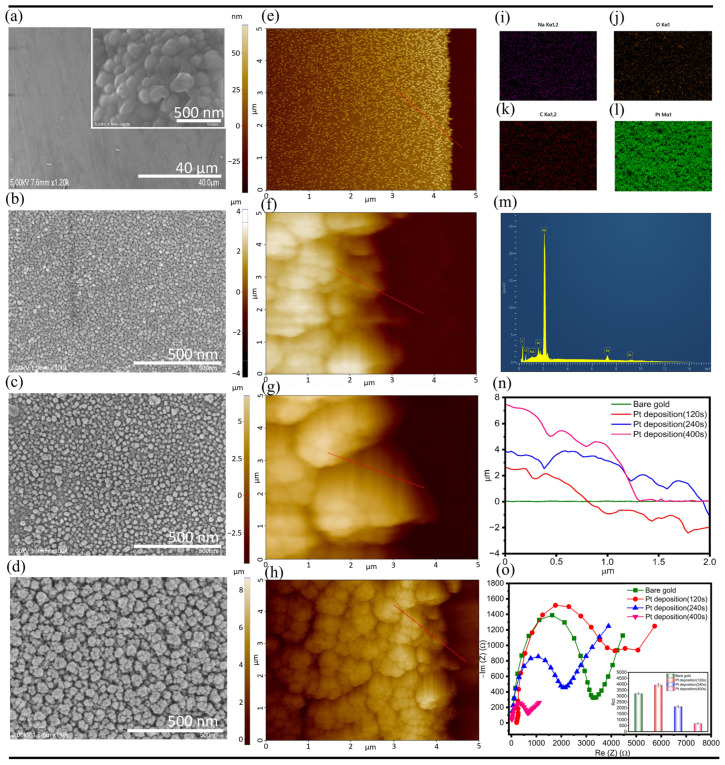
Morphological and electrochemical characterization of Pt-black electrodeposition on the IWEA platform. (**a**) SEM image of the pristine gold IWEA. (**b**–**d**) SEM images illustrating Pt-black formation on the IWEA after electrodeposition for 120 s, 240 s, and 400 s, respectively. (**e**) AFM image representing the surface topology of the pristine gold IWEA. (**f**–**h**) AFM images of Pt-black coated IWEA obtained at 120 s, 240 s, and 400 s deposition intervals. The red lines indicate the line profiles used for the quantitative surface height analysis. (**i**–**m**) Elemental mapping via EDS demonstrates a consistent distribution of Pt-black across IWEA. (**n**) Quantitative AFM surface analysis showing increased height variation with longer deposition times. (**o**) Nyquist plot and charge-transfer resistance values (shown in the inset) of Pt-black-modified IWEA at different deposition times, highlighting enhanced electron-transfer kinetics, particularly for the 400 s deposition sample.

SEM analysis revealed a distinct evolution in surface morphology with increasing deposition time. The bare Au electrode displayed a smooth and featureless surface (Figure 4a), whereas initial Pt nucleation sites became visible after 120 s of deposition (Figure 4b). At 240 s, the Pt nanoclusters grew and partially interconnected, forming a more continuous porous network (Figure 4c). Increasing the deposition time to 400 s generated a dense cauliflower-like Pt-black architecture (Figure 4d), a morphology widely associated with enlarged electroactive surface area and accelerated electron-transfer kinetics [42]. AFM images (Figure 4e–h) additionally supported the surface modification following Pt-black deposition. The AFM topography reveals a progressive increase in surface height variation as the deposition time increases, indicating that the surface becomes more structurally developed at 400 s compared to the shorter deposition conditions, as illustrated in Figure 4n. The successful deposition of Pt-black was further confirmed by EDS elemental mapping (Figure 4i–m), which clearly demonstrated a uniform distribution of Pt-black across the electrode surface.

Electrochemical impedance spectroscopy (Figure 4o) with the corresponding charge-transfer resistance (Rct) values shown in the inset further correlated with the morphological observations. A deposition time of 400 s resulted in the lowest Rct value, corresponding to an approximately 78.7 ± 7.1% reduction compared to the bare IWEA. The significant decrease in Rct indicates enhanced interfacial electron-transfer efficiency due to the highly porous Pt-black nanostructure. In contrast, shorter deposition durations resulted in incomplete Pt coverage and relatively higher Rct values, while excessive Pt growth may lead to nanoparticle agglomeration and reduced electrochemical performance. These findings highlight the importance of controlled Pt-black electrodeposition in achieving highly conductive and electrochemically active sensing interfaces.

### 3.2. Bacterial Detection

**Figure 5:**
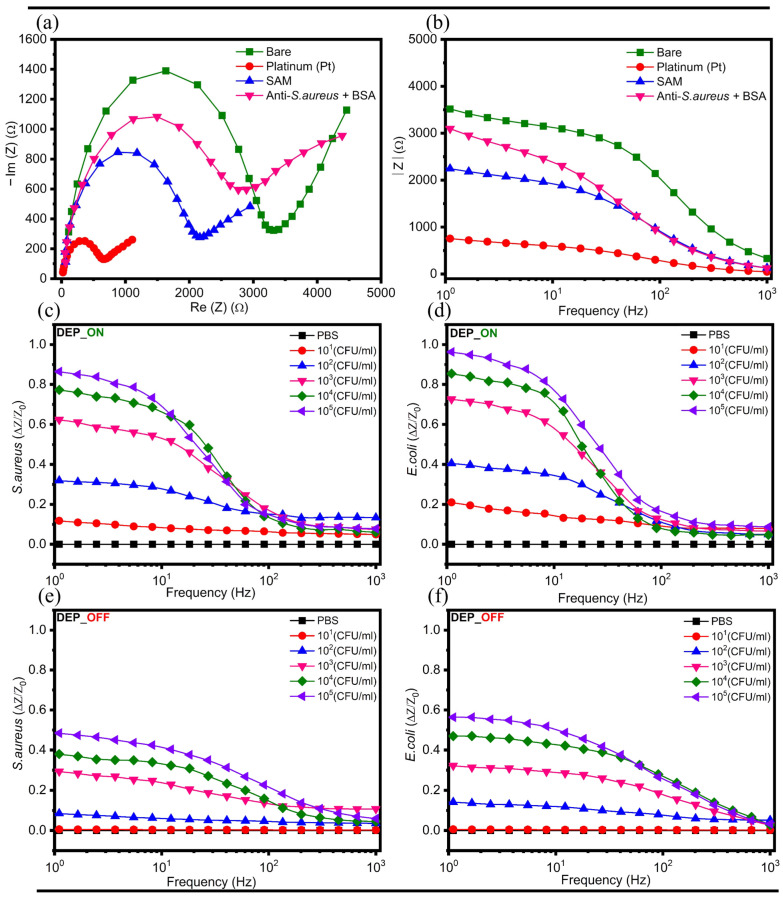
Stepwise surface functionalization and bacterial detection performance of the IWEA. (**a**) Nyquist plots illustrating stepwise surface modification of the IWEA (bare Au, Pt-black, SAM, Anti-*S. aureus*, and BSA) in 5 mM [Fe(CN)_6_]^3−^/^4−^. (**b**) Corresponding Bode plots for each functionalization step. (**c**,**d**) Impedance responses (ΔZ/Z_0_) of *S. aureus* and *E. coli* across 10–10^5^ CFU/mL under DEP-assisted detection. (**e**,**f**) Impedance responses (ΔZ/Z_0_) of *S. aureus* and *E. coli* across 10–10^5^ CFU/mL under non-DEP passive detection.

Prior to bacterial detection and antibiotic susceptibility measurements, electrochemical impedance spectroscopy (EIS) was performed in 5 mM [Fe(CN)_6_]^3−^/^4−^ solution over a frequency range of 1 Hz to 1 kHz to evaluate the sequential surface modification of the developed interdigitated wave electrode array (IWEA). The corresponding Nyquist and Bode plots shown in Figure 5a,b demonstrate the impedance variations associated with each functionalization step. Initially, the bare gold IWEA exhibited high charge transfer resistance (Rct). Following Pt-black deposition, a pronounced decrease in Rct was observed, which can be attributed to the enhanced electrical conductivity and enlarged electroactive surface area introduced by the nanostructured Pt-black layer. Subsequent formation of the self-assembled monolayer (SAM) resulted in an increase in Rct due to its insulating nature, which hindered electron transfer at the electrode–electrolyte interface. Further immobilization of the *S. aureus* antibody and BSA blocking produced an additional increase in impedance, confirming successful surface functionalization, as shown in Figure 5a. The same trend was observed in the Bode spectra, shown in Figure 5b, where impedance magnitude decreased after Pt-black modification and gradually increased following successive SAM formation, antibody immobilization, and BSA blocking steps.

The developed IWEA platform was evaluated for bacterial detection using a combined impedance–DEP sensing strategy employing *S. aureus* and *E. coli* as Gram-positive and Gram-negative bacteria, respectively. Bacterial suspensions prepared in 0.1× PBS at concentrations ranging from 10 to 10^5^ CFU/mL were introduced onto the sensing region of the IWEA. Subsequently, a dielectrophoretic (DEP) force was applied using a 100 kHz AC electric field at 10 V_pp_ for 30 min to selectively enrich highly polarizable bacterial cells at regions of intensified electric-field distribution while minimizing joule heating and preventing cell damage [43]. Following DEP enrichment, the electric field was switched off, and impedance measurements were immediately performed at a low excitation voltage for bacterial detection analysis. As shown in Figure 5c,d, both *S. aureus* and *E. coli* exhibited concentration-dependent impedance responses across the investigated range, with ΔZ/Z_0_ values increasing with increasing bacterial concentration. To assess the role of dielectrophoretic (DEP) enrichment, control experiments were performed in the absence of the applied DEP field, as shown in Figure 5e,f. Under non-DEP conditions, significantly reduced impedance responses were observed, and the lowest bacterial concentration (10 CFU/mL) was not reliably distinguishable from the bacteria-free control.

**Figure 6:**
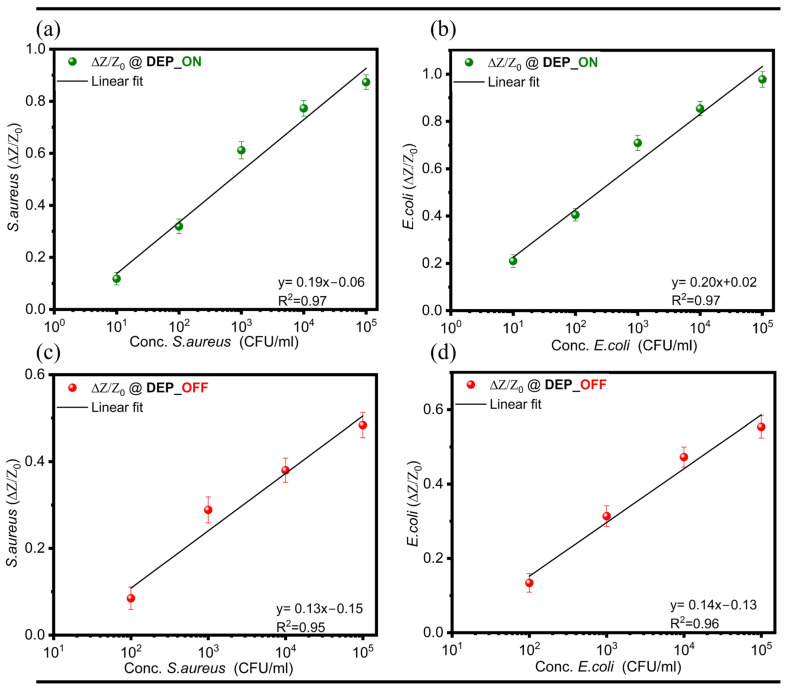
Calibration plots illustrating impedance responses (ΔZ/Z_0_) obtained at 1 Hz for bacterial detection under DEP-assisted and non-DEP passive operation. (**a**,**b**) Impedance responses (ΔZ/Z_0_) of *S. aureus* and *E. coli* at (10–10^5^ CFU/mL) under DEP-assisted operation. (**c**,**d**) Impedance responses (ΔZ/Z_0_) of *S. aureus* and *E. coli* at (10^2^–10^5^ CFU/mL) under non-DEP passive operation. Data are presented as mean values (*n* = 3), with error bars indicating the standard deviation.

The bacterial detection capability under DEP-assisted and non-DEP passive conditions was evaluated by generating calibration curves based on the impedance change (ΔZ/Z_0_) measured at 1 Hz as a function of bacterial concentration, as shown in Figure 6a–d. Under DEP-assisted conditions, *S. aureus* and *E. coli* exhibited strong linear correlations across a broad concentration range of 10–10^5^ CFU/mL. The corresponding linear regression equations were y = 0.19x − 0.06 (R^2^ = 0.97) and y = 0.20 x + 0.02 (R^2^ = 0.97), respectively, as presented in Figure 6a,b. In contrast, measurements obtained under non-DEP passive detection conditions showed linear responses only within the concentration range of 10^2^–10^5^ CFU/mL. The regression equations for *S. aureus* and *E. coli* were determined to be y = 0.13x − 0.15 (R^2^ = 0.95) and y = 0.14x − 0.13 (R^2^ = 0.96), respectively, as shown in Figure 6c,d. These results indicate that the integrated impedance–DEP sensing strategy promotes local enrichment of bacterial cells at the sensing interface, leading to improved sensitivity and lower detection limits, especially at low bacterial concentrations.

### 3.3. Antibiotic Susceptibility Testing: Impedance Responses, Scanning Electron Microscopy, and Disk Diffusion Analysis

Our developed interdigitated wave electrode array (IWEA) was employed to investigate antibiotic-induced changes in bacterial viability using impedance measurements. For this purpose, three antibiotics, namely vancomycin, gentamicin and ampicillin, were selected to evaluate antibiotic susceptibility in two representative bacterial strains: *S. aureus* (Gram-positive) and *E. coli* (Gram-negative). The experiments were performed in two sequential stages. Initially, bacterial samples of *S. aureus* and *E. coli* were incubated with varying concentrations of the selected antibiotics for 1 h, including a control sample without antibiotics. This approach enabled the evaluation of antibiotic-induced effects across a range of bacterial viabilities, from fully viable to partially and fully inhibited conditions. Subsequently, the interdigitated wave electrode array (IWEA) was cleaned with deionized (DI) water and 70% (*v*/*v*) ethanol, followed by drying under nitrogen. A defined volume (700 ± 5.0 µL) of the antibiotic-treated bacterial suspension (10^5^ CFU/mL for both *S. aureus* and *E. coli*) was then exposed to the electrode surface, where a dielectrophoretic (DEP) force (100 kHz) was applied at 10 V_pp_ for 30 min to selectively concentrate viable cells at regions of high electric-field intensity. Following DEP enrichment, the electric field was turned off, and impedance measurements were immediately performed at a low excitation voltage to monitor antibiotic-induced changes in bacterial viability. The impedance response remained stable after termination of the DEP field, suggesting no loss of captured cells from the electrode surface during subsequent measurements. The impedance response was expressed as the differential impedance change (ΔZ/Z_0_) relative to the initial impedance values.

**Figure 7:**
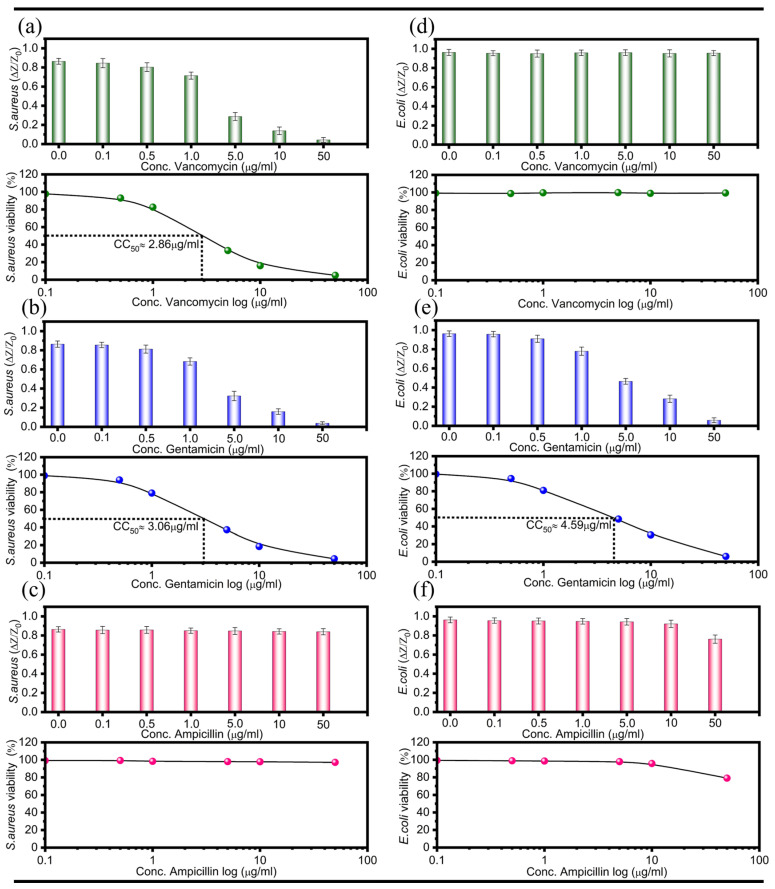
Impedance responses (ΔZ/Z_0_) and corresponding CC_50_ values for antibiotic-treated *S. aureus* and *E. coli*. (**a**–**c**) Concentration-dependent impedance responses of *S. aureus* following treatment with vancomycin, gentamicin, and ampicillin, respectively, along with extracted CC_50_ values where applicable. (**d**–**f**) Impedance responses of *E. coli* under treatment with vancomycin, gentamicin, and ampicillin, respectively, along with CC_50_ analysis where applicable. Antibiotic-susceptible bacteria exhibit a pronounced concentration-dependent decrease in ΔZ/Z_0_ compared to untreated controls, enabling CC_50_ estimation, whereas resistant bacteria show minimal or no impedance variation, resulting in no meaningful CC_50_ response.

Figure 7a–f present the differential impedance responses (ΔZ/Z_0_) and corresponding viability curves and estimated CC_50_ values obtained for *S. aureus* and *E. coli* treated with different concentrations of vancomycin, gentamicin, and ampicillin. For susceptible bacterial–antibiotic combinations, a concentration-dependent decrease in ΔZ/Z_0_ values was observed with increasing antibiotic concentration, indicating reduced bacterial viability. In contrast, bacterial–antibiotic combinations exhibiting resistant or weakly affected conditions showed impedance responses comparable to the untreated control, indicating minimal or no changes in bacterial viability, even at higher antibiotic concentrations. The CC_50_ value represents the antibiotic concentration required to reduce bacterial viability by 50%, as estimated from the impedance-derived dose–response curves.

In the case of *S. aureus*, significant reductions in ΔZ/Z_0_ values were observed following treatment with vancomycin and gentamicin, demonstrating susceptibility toward these antibiotics, with estimated CC_50_ values of 2.86 ± 0.22 µg/mL and 3.06 ± 0.20 µg/mL, respectively, as shown in Figure 7a,b. However, ampicillin-treated *S. aureus* exhibited stable impedance responses similar to the control condition, indicating resistance to ampicillin, as shown in Figure 7c. Similarly, *E. coli* exhibited negligible impedance variation upon vancomycin exposure, consistent with the intrinsic resistance of Gram-negative bacteria to vancomycin, as depicted in Figure 7d [44]. Conversely, gentamicin-treated *E. coli* samples exhibited a clear concentration-dependent decrease in ΔZ/Z_0_ values, with an estimated CC_50_ value of 4.59 ± 0.30 µg/mL, indicating strong antibacterial activity, as displayed in Figure 7e. Ampicillin-treated *E. coli* samples showed much smaller reductions in impedance response, suggesting limited or partial antibacterial effectiveness within the tested concentration range, as shown in Figure 7f.

The observed impedance variations were closely associated with viability-dependent dielectrophoretic enrichment of bacterial cells at the sensing interface. Following antibiotic treatment, bacterial cells that retained physiological activity were preferentially attracted and concentrated near regions of high electric-field intensity under the applied DEP field, resulting in measurable impedance changes. As antibiotic concentration increased, susceptible bacterial populations exhibited reduced viability, leading to decreased DEP-assisted accumulation at the sensing region and consequently lower impedance responses. In contrast, bacterial populations maintaining viability or showing resistance after antibiotic exposure continued to exhibit stable impedance signals due to effective dielectrophoretic retention near the electrode surface. Collectively, these findings demonstrate the potential of our developed interdigitated wave electrode array (IWEA) integrated with viability-dependent DEP enrichment of bacterial cells as a rapid and effective platform for real-time antibiotic susceptibility monitoring.

**Figure 8:**
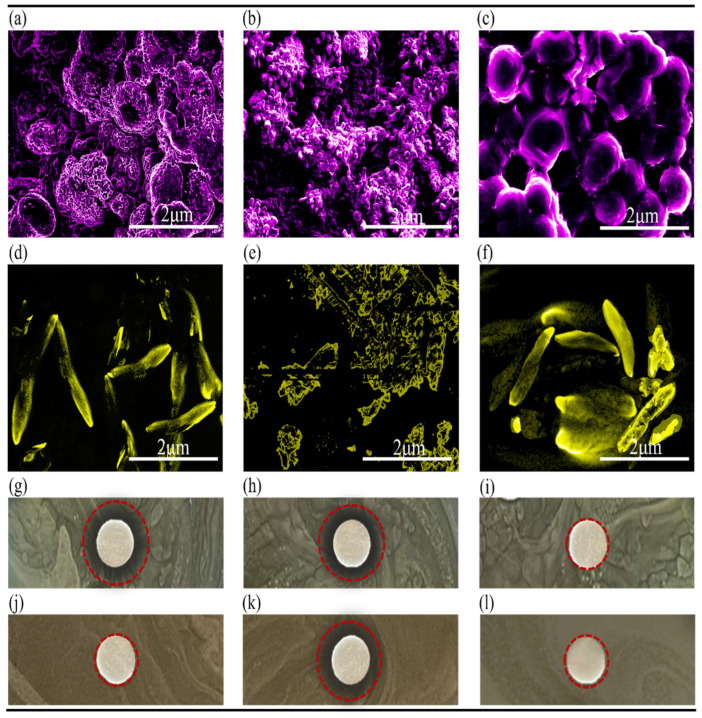
Scanning electron microscopy (SEM) images of *S. aureus* treated with (**a**) vancomycin, (**b**) gentamicin, and (**c**) ampicillin, and *E. coli* treated with (**d**) vancomycin, (**e**) gentamicin, and (**f**) ampicillin. Corresponding optical images of disk diffusion assays for *S. aureus* (**g**–**i**) and *E. coli* (**j**–**l**) against vancomycin, gentamicin, and ampicillin, respectively. The red circles indicate the zones of inhibition. Antibiotic-susceptible bacteria exhibit pronounced morphological disruption and cellular deformation in SEM images, accompanied by distinct zones of inhibition in disk diffusion assays. In contrast, resistant bacteria retain intact morphology with no observable inhibition zones.

To further validate the impedance-based antibiotic susceptibility results, morphological changes in bacterial cells following antibiotic treatment were examined through scanning electron microscope (SU8600, Hitachi, Tokyo, Japan). Representative SEM images were acquired for selected bacterial–antibiotic combinations exhibiting either susceptible or resistant responses in the impedance measurements, as shown in Figure 8a–f, rather than for all tested antibiotic concentrations. The SEM analysis demonstrated that bacterial–antibiotic combinations exhibiting impedance responses identical to the untreated bacteria or control samples retained well-defined cellular morphology and preserved membrane integrity, indicating resistance toward the corresponding antibiotic treatment. In contrast, bacterial–antibiotic combinations associated with significant reductions in ΔZ/Z_0_ values exhibited pronounced structural deformation, membrane disruption, and collapsed cellular morphology, confirming antibiotic-induced bacterial damage. Specifically, ampicillin-treated *S. aureus* cells (Figure 8c) and vancomycin- or ampicillin-treated *E. coli* cells (Figure 8d,f) exhibited intact morphologies consistent with their resistant or weakly affected impedance responses. Conversely, vancomycin- and gentamicin-treated *S. aureus* samples (Figure 8a,b), as well as gentamicin-treated *E. coli* samples (Figure 8e), showed severe morphological damage corresponding to the reduced impedance responses. These observations further confirm that the impedance responses obtained using the developed IWEA platform are strongly associated with viability-dependent DEP enrichment and antibiotic-induced structural changes in bacterial cells.

To further benchmark the developed platform against a conventional antibiotic susceptibility testing method, disk diffusion assays were additionally performed. The inhibition zone patterns obtained from the disk diffusion assays showed strong agreement with the impedance responses and SEM analysis. *S. aureus* exhibited clear inhibition zones against vancomycin and gentamicin (Figure 8g,h), consistent with the significant reductions observed in the corresponding ΔZ/Z_0_ values and morphological damage in SEM analysis. In contrast, ampicillin-treated *S. aureus* showed no distinct inhibition zone (Figure 8i), together with stable impedance responses and preserved cellular morphology, indicating resistance to ampicillin treatment. Similarly, *E. coli* showed pronounced susceptibility toward gentamicin with a distinct inhibition zone (Figure 8k), while vancomycin- and ampicillin-treated *E. coli* samples exhibited comparatively negligible or weaker inhibition behavior (Figure 8j,l), consistent with the corresponding impedance and SEM results. The measured inhibition zone diameters are summarized in Table 1, where zero or negligible inhibition zones indicate bacterial resistance.

## 4. Conclusions

This study presents a real-time, label-free interdigitated wave electrode array (IWEA) that integrates dielectrophoretic (DEP) enrichment with impedance spectroscopy for rapid bacterial detection and AST. Finite element simulations demonstrated that the IWEA geometry generated enhanced electric-field localization and a 33.84% increase in average square electric field (E^2^) compared to the conventional IDEA, promoting improved DEP-based bacterial enrichment. Based on the simulation results, the modeled IWEA was selected for fabrication, followed by surface modification with Pt-black to enhance electrochemical activity and subsequent antibody immobilization to enable selective bacterial detection. The developed platform enabled sensitive detection of both Gram-positive (*S. aureus*) and Gram-negative (*E. coli*) bacteria in 0.1× PBS across (10–10^5^ CFU/mL) within 30 min using DEP-assisted preconcentration (100 kHz, 10 V_pp_), demonstrating superior performance over passive non-DEP operation.

The developed IWEA was also successfully used for AST, where antibiotic-induced changes in bacterial viability were rapidly translated into differential impedance responses following DEP enrichment. Susceptible bacterial exhibited concentration-dependent reductions in impedance response, whereas resistant bacteria maintained impedance behavior similar to untreated controls. Quantitative evaluation yielded CC_50_ values of 2.86 ± 0.22 µg/mL and 3.06 ± 0.20 µg/mL for vancomycin and gentamicin against *S. aureus*, respectively, and 4.59 ± 0.30 µg/mL µg/mL for gentamicin against *E. coli*. Importantly, the impedance-based findings were in strong agreement with SEM imaging and disk diffusion assays, confirming the reliability of the proposed sensing strategy.

Overall, the proposed DEP-integrated IWEA platform provides a rapid, sensitive, and label-free strategy for simultaneous bacterial detection and AST, significantly reducing analysis time compared to conventional culture-based and impedance-only methods. Owing to its multiplexing capability, low sample requirement, portability, and real-time monitoring compatibility, the developed system shows strong potential for point-of-care diagnostics, antimicrobial screening, and AMR management.

## Figures and Tables

**Table 1:** Antibacterial activity expressed as inhibition zone diameters (mm) for *S. aureus* and *E. coli* against vancomycin, gentamicin, and ampicillin using disk diffusion method.

Inhibition Zone (mm)			
Bacteria/Antibiotics	Vancomycin	Gentamicin	Ampicillin
*S. aureus*	18.60 ± 0.50	16.20 ± 0.50	0.00 ± 0.00
*E. coli*	0.00 ± 0.00	20.30 ± 0.50	0.00 ± 0.00

## Data Availability

The original contributions presented in this study are included in the article. Further inquiries can be directed to the corresponding author.
